# Informed Consent in Epidemiologic Research before the Implementation of Ethical Guidelines

**DOI:** 10.2188/jea.14.177

**Published:** 2005-03-18

**Authors:** Akiko Tamakoshi

**Affiliations:** 1 Department of Preventive Medicine, Nagoya University Graduate School of Medicine, 65 Tsurumai-cho, Showa-ku, Nagoya 466-8550, Japan.

**Keywords:** Ethics, Human Experimentation, Epidemiology, Informed Consent, Guidelines

## Abstract

On June 17, 2002, the Ministry of Education, Culture, Sports, Science and Technology, together with the Ministry of Health, Labour and Welfare, introduced the Ethical Guidelines for Epidemiological Research. Although studies begun before the adoption of the Guidelines are not necessarily required to conform to them, some studies have been reviewed anew by ethics review committees. In this article for the Young Investigator Award of the Japan Epidemiological Association, therefore, the author would like to offer an overview of informed consent in epidemiologic researches conducted before the introduction of the Guidelines. It is hoped that this may serve as a reference as to the contents and status of ethical considerations in these studies, for use in examinations of research that was already in progress when the Guidelines were introduced.

Since the Declaration of Helsinki,^1^ the importance of informed consent in medical and epidemiologic studies has come to be recognized around the world. In 1991, the Council for International Organizations of Medical Sciences (CIOMS) prepared the International Guidelines for Ethical Review of Epidemiological Studies specifically for epidemiologic research.^2^ Japan was slower in introducing such guidelines, but on June 17, 2002 the Ministry of Education, Culture, Sports, Science and Technology and the Ministry of Health, Labour and Welfare, introduced the Ethical Guidelines for Epidemiological Research.^3^ In the Guidelines, the principles for informed consent differ according to the study design and materials used. Studies begun after the introduction of the guidelines are obviously required to conform to them. On the other hand, studies underway at the time when the Guidelines were implemented do not necessarily need to conform to them, but many epidemiologic studies, particularly cohort studies, extend over a long period of time. Thus, the judgments of ethics review committees have been requested for some studies that were underway, and in more than a few cases the committees advised that re-consent was needed in the light of current standards. The author reports the results of a fact-finding survey on informed consent of researchers before the Guidelines were introduced, classified according to the Guidelines.

This article was written on the occasion that the author received the Young Investigator Award of the Japan Epidemiological Association in 2003. The author is involved in conducting a large-scale cohort study, and at the same time actively seeks to build better relations between society and the research world.

## METHODS

The subjects were Japanese epidemiologic researchers who had presented research in the form of an academic conference report or written paper. They were requested to complete an anonymous questionnaire regarding informed consent and other matters in the study that they had presented. The subjects were selected at random from researchers who had been corresponding authors in papers published in 1997 in the Japanese Journal of Public Health, Journal of Occupational Health, Japanese Journal of Hygiene, International Journal of Epidemiology, American Journal of Epidemiology, or Journal of Epidemiology; or who had been the chief presenter of a study with human subjects among academic conference reports to the Japan Epidemiological Association, Japanese Society for Hygiene, or Japanese Society of Public Health in 1997, or the Japan Epidemiological Association or Japan Society for Occupational Health in 1998. When two or more of the above cases applied to a single researcher, priority was given first to the most recent presentation and second to written papers. Each researcher was limited to one report, and in the end there were a total of 490 subjects. The questionnaire contained items on study attributes (design, materials, etc.); subject attributes (data source, population, etc.); whether or not subjects were given an explanation of the study, and if so the method and content of the explanation; and whether or not consent was received and if so the method and content of the consent. The questionnaires were sent on September 2, 1998, and a second written request was sent to subjects for whom it was unclear whether a response had been received.

## RESULTS

Responses were received from 330 subjects by the end of November 1998. Excluding 11 questionnaires that could not be delivered because of unknown addresses or other reasons, the response rate was 69%. The response rate for journal papers (74%) was higher than that for conference presentations (67%). Given the possibility that authors of studies that did not provide explanations or obtain consent may have been less likely to respond, the actual explanation and consent rates during this period may be somewhat lower than the results.

Information on the study was considered to have been given if a respondent answered “yes” to the question: “Was an explanation of the study given in some form to the subjects or the proxies for the subjects?” Table 1 shows the aggregate results for explanations of study design and materials used. Explanations of some type were given in 71% of all studies. Explanation was given in 86% of interventional studies that used human biological specimens, in 80% of interventional studies that did not use human biological specimens, in 63% of cohort studies, and in 74% of observational studies other than cohort studies.

**Table 1.** Percentage of studies in which informed consent was obtained.

Study design Information used	interventional studies				observational studies										total

					cohort studies					other than cohort studies					
		
	human biological specimen		others	total	human biological specimen		others		total	human biological specimen		others		total	
				
	genome	others			genome	others	newly obtained	existing materials		genome	others	newly obtained	existing materials		
Give information on the study to subjects															
yes	1	5	12	18	2	11	28	15	56	15	49	139	77	284	233
	(100)	(83)	(80)	(82)	(67)	(58)	(76)	(54)	(63)	(94)	(79)	(85)	(56)	(74)	(71)
no	0	1	3	4	1	8	9	13	33	1	12	23	58	94	93
		(17)	(20)	(18)	(33)	(42)	(24)	(46)	(37)	(6)	(19)	(14)	(42)	(24)	(28)

Obtain consent form subjects															
yes	1	5	10	16	2	8	21	10	41	14	33	90	49	190	156
	(100)	(83)	(67)	(73)	(67)	(42)	(57)	(36)	(46)	(88)	(53)	(55)	(36)	(49)	(47)
no	0	1	4	5	1	10	15	15	42	2	26	68	76	172	152
		(17)	(27)	(23)	(33)	(53)	(41)	(54)	(47)	(13)	(42)	(41)	(55)	(45)	(46)

Total	1	6	15	22	3	19	37	28	89	16	62	164	138	384	330
	(100)	(100)	(100)	(100)	(100)	(100)	(100)	(100)	(100)	(100)	(100)	(100)	(100)	(100)	(100)

Percentages in parentheses

Consent was considered to have been given if a respondent answered “yes” to the question: “Was consent obtained in some form from subjects or their proxies?” By this criterion, consent was obtained in 47% of all studies. Consent was obtained in 73% of all interventional studies and 86% of interventional studies in which human biological samples were used, in 46% of cohort studies, and in 50% of other observational studies. As shown in Table 2, consent was obtained in written form in 28% of studies in which consent was obtained, and opt in participation was adopted in 36% of studies.

**Table 2.** Procedures for obtaining consent.

Study design Information used	interventional studies				observational studies										total

					cohort studies					other than cohort studies					
		
	human biological specimen		others	total	human biological specimen		others		total	human biological specimen		others		total	
				
	genome	others			genome	others	newly obtained	existing materials		genome	others	newly obtained	existing materials		
Means of consent															
oral consent	1	3	6	12	0	3	10	4	17	6	15	43	32	98	80
	(100)	(60)	(60)	(75)		(38)	(48)	(40)	(41)	(43)	(45)	(48)	(65)	(52)	(51)
written consent	0	2	4	6	2	5	8	4	19	7	13	24	6	49	43
		(40)	(40)	(38)	(100)	(63)	(38)	(40)	(46)	(50)	(39)	(27)	(12)	(26)	(28)
others	0	0	0	0	0	0	3	2	5	0	4	22	10	39	31
							(14)	(20)	(12)		(12)	(24)	(20)	(21)	(20)
Type of participation															
opt in	0	4	6	10	1	3	5	2	10	4	12	36	14	67	56
		(80)	(60)	(63)	(50)	(38)	(24)	(20)	(24)	(29)	(36)	(40)	(29)	(35)	(36)
opt out	1	1	4	6	1	5	15	7	29	9	19	52	32	115	94
	(100)	(20)	(40)	(38)	(50)	(63)	(71)	(70)	(71)	(64)	(58)	(58)	(65)	(61)	(60)

Total	1	5	10	16	2	8	21	10	41	14	33	90	49	190	156
	(100)	(100)	(100)	(100)	(100)	(100)	(100)	(100)	(100)	(100)	(100)	(100)	(100)	(100)	(100)

Percentages in parentheses

## DISCUSSION

In the Guidelines,^3^ epidemiologic research was classified as shown in Table 3, and a distinction is made in the method of informed consent. However, studies that include gene analysis are subject not to the epidemiologic guidelines but to the Ethics Guidelines for Human Genome/Gene Analysis Research established on March 29, 2001 by the following 3 Ministries of the Japanese government; the Ministry of Education, Culture, Sports, Science, and Technology; the Ministry of Health, Labour and Welfare; and the Ministry of Economy, Trade and Industry.^4^ In this paper, following the divisions in the Ethical Guidelines for Epidemiological Research, it is possible to determine whether the intervention studies included gene analysis or whether or not they used some other type of human biological specimens. Only one study included gene analysis, and in it procedures for explanation and consent were adopted. However, this study used an opt out style of participation. Current studies that include gene analysis are required to follow the genome guidelines,^4^ according to which “A Group B human specimen (a human specimen on which consent has been obtained at the time of provision, but only for research that does not articulate its use in human genome/gene analysis research) or a Group C human specimen (a human specimen for which consent for its use in research has not been obtained) may not, in principle, be used in human genome/gene analysis research unless consent from a donor, proxy consenter or equivalent person is newly obtained in accordance with the process etc. prescribed in the present Guidelines.” Therefore, if biological specimens obtained in 1998 are used in further research, it is necessary in many cases to obtain the judgment of an ethics review committee with regard to re-consent, the possibility of maintaining anonymity, and whether or not the requirements have been met. As of 1998, some interventional studies that include use of human biological specimens without gene analyses had not adopted procedures for informed consent, but the current Guidelines state that “Informed consent shall be, in principle, obtained from all research subjects.”

**Table 3.** Procedure for obtaining informed consent according to the Guidelines.

type of epidemiologic research	informed consent required
(1) Interventional studies	
1) Research using human biological specimens	
A. In the event specimens are collected by invasive methods (e.g., venipuncture)	Written informed consent is needed.
B. In the event specimens are collected by non-invasive methods	Informed consent is needed, but does not necessarily have to be in writing.
2) Research not using human biological specimens	
A. In the event interventional research is focused on individual subjects	Informed consent is needed, but does not necessarily have to be in writing.
B. In the event interventional research is focused on an entire population	Opportunity to refuse inclusion in the research is needed.

(2) Observational studies	
1) Research using human biological specimens	
A. In the event specimens are collected by invasive methods	Written informed consent is needed.
B. In the event specimens are collected by non-invasive methods	Informed consent is needed, but does not necessarily have to be in writing.
2) Research not using human biological specimens	
A. Observational research using data other than existing materials	Opportunity to refuse inclusion in the research is needed.
B. Observational research using only existing materials	Publishing all relevant details regarding the study is required.

In interventional studies that do not use biological specimens, explanation and consent is approached differently depending on whether the units are individuals or groups. In the case of individual units, written consent is not necessary but it is necessary to obtain consent individually. For group units, it is necessary to guarantee an opportunity for refusal. In 1998 surveys, the target units for explanation and consent were unclear in studies that did not use biological specimens. However, in cases when the research placed a burden on subjects (as a result of the intervention) it is difficult to believe that absolutely no explanation was given. Therefore, this was probably done in group units. At that time, information on research was generally not disclosed, but today with the spread of the Internet and other means of communication it is expected that such disclosure will become easier.

The Guidelines state that in observational studies it is necessary to explain the study and obtain consent when biological specimens are used, but oral consent is sufficient if the procedure is not invasive. Dividing studies into cohort studies that may extend long-term and other studies, a tendency was seen for there to be no explanation and consent in cohort studies. This may be related to the fact that many cohorts were formed at medical checkup or other sites, without the subjects being aware that they were involved in research. Studies that do not use biological specimens are divided into those that use new materials and those that use existing materials. If new materials are to be used it is necessary to disclose information and ensure subjects an opportunity to refuse participation. If existing materials are to be used, information disclosure alone is sufficient. In observational studies other than cohort studies in the present survey, an explanation was provided in 85% of cases and consent obtained in 55% of cases in which new materials were to be used.

The Guidelines state that “Epidemiological studies which were started prior to the implementation of the Guidelines will be exempted, nonetheless fair compliance with these Guidelines is advocated.” Thus, even for studies started before the implementation of the Guidelines, a request may be made for judgment by an ethics review committee at such times as when new research analysis is done. In cohort studies in particular, continuous data gathering is needed, and/or a long period of time is required for follow-up, so it is very likely that procedures for an ethical judgment will be adopted. In such cases, initial explanation and consent procedures will be compared with current standards, and, if the ethics review committee judges they are insufficient, procedures for re-consent may be adopted. However, as shown in this paper, before the establishment of the Guidelines, consent was not obtained in about a half of studies, and even if it was obtained, in most cases it was obtained orally and in an opt out type of study. While it may not be possible to generalize, after carefully considering the purpose and content of the respective study, it would seem acceptable in many cases to proceed with the study without procedures for re-consent if consideration is given to anonymity and appropriate precautions are taken at the time of publication.

The most common reason given by researchers both for not providing an explanation of the study and not obtaining consent was “The study was in no way detrimental to the subjects, so we did not consider there to be an ethical problem.” Most epidemiologic studies are rooted in actual public health activities, so it is difficult to distinguish between the public health activity and the research. Moreover, because there is little invasive burden, the attitude represented by the above statement was probably the general attitude of researchers before the establishment of the Guidelines. The Guidelines state the need for a judgment by a third-party organization (ethics review committee), and today we expect to seen an increasing number of studies that proceed with the general consensus of society.

Two years have passed since the implementation of the Guidelines. It is expected that there will be an increasing number of studies that truly respect the human rights of the subjects, which is at the foundation of the Guidelines.
